# Co-expression of cellulase and xylanase genes in *Sacchromyces cerevisiae* toward enhanced bioethanol production from corn stover

**DOI:** 10.1080/21655979.2019.1682213

**Published:** 2019-10-29

**Authors:** Wenjing Xiao, Huanan Li, Wucheng Xia, Yuxian Yang, Pan Hu, Shanna Zhou, Yanmei Hu, Xiaopeng Liu, Yujun Dai, Zhengbing Jiang

**Affiliations:** a State Key Laboratory of Biocatalysis and Enzyme Engineering, School of Life Sciences, Hubei University, Wuhan, PR China; b Hubei Key Laboratory of Industrial Biotechnology, School of Life Science, Hubei University, Wuhan, PR China; c Department of Biological Science and Technology, Hubei University for Nationalities, Ensi, P. R. China; d Hubei Province Research Center of Engineering Technology for Utilization of Botanical Functional Ingredients, Hubei Engineering University, Xiaogan, P. R. China

**Keywords:** Cellulase, xylanase, co-expression, lignocellulose, corn stover

## Abstract

Lignocellulose is considered as a good resource for producing renewable energy. Previous *in vitro* studies have shown the synergistic action between cellulase and xylanase during lignocellulose biohydrolysis. In order to achieve the same effect in *S. cerevisiae* to enhance the practical biotransformation, two recombinant *Saccharomyces cerevisiae* strains (*INVSc1*-CBH-CA and *INVSc1*-CBH-TS) with co-expressed cellulase and xylanase were constructed. The cellulase and xylanase activities in *INVSc1*-CBH-CA and *INVSc1*-CBH-TS were 716.43 U/mL and 205.13 U/mL, 931.27 U/mL and 413.70 U/mL, respectively. The recombinant *S. cerevisiae* can use the partly delignified corn stover (PDCS) more efficiently and more ethanol producted than *S. cerevisiae* only expressing cellulase. Fermentation with *INVSc1*-CBH-CA and *INVSc1*-CBH-TS using PDCS ethanol yields increased by 1.7 and 2.1 folds higher than *INVSc1*-CBH, 2.8 and 3.4 folds higher than the wild type *S. cerevisiae*. The strategy of co-expression cellulase and xylanase in *saccharomyces cerevisiae* is effective and can be *a* foundation to research the mechanism of synergy effect of cellulose and xylanase.

## Graphical Abstract

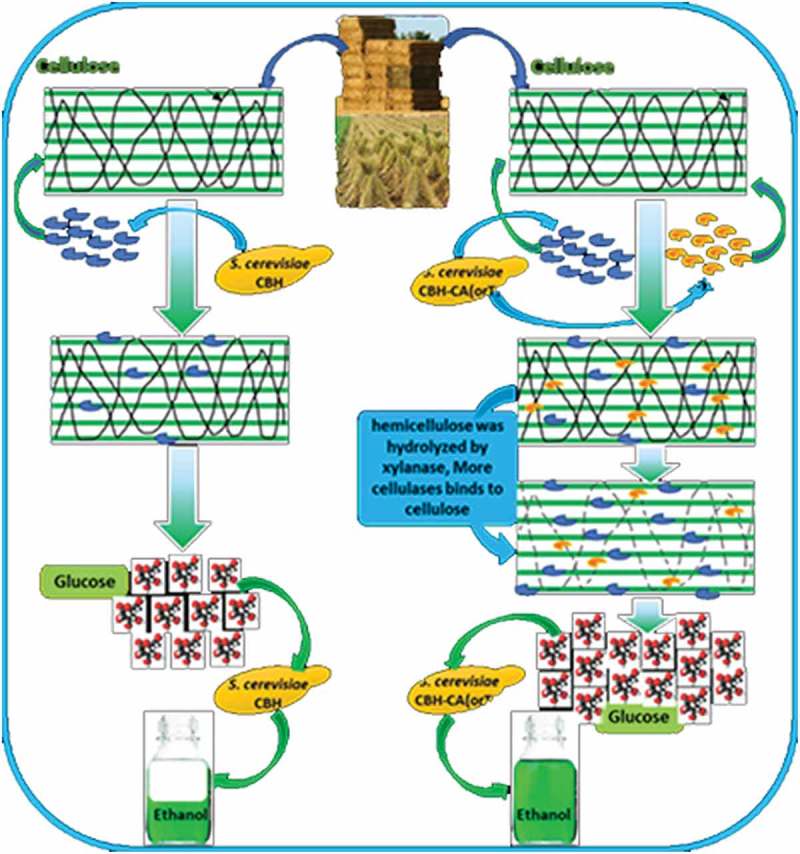

**Abbreviations:** CA, the xylanase gene *CA*; CBH, the cellulase gene *CBH*; CBP, consolidated bioprocessing; CMC, carboxymethyl cellulose; DNS, dinitrosalicylic acid; LB, lysogeny broth; OD_600_, optical density at 600 nm; SC, synthetic complete; SSF, the synchronous saccharification and fermentation; TS, the xylanase gene *TS*; YPD, the yeast extract peptone dextrose.

## 1. Introduction

Lignocellulose is a major constituent of plant biomass and consists mainly of cellulose, hemicellulose, and lignin etc [1,2]. It is widely distributed and can be easily obtained from wood, weeds, and agricultural residues, etc [3]. Because of abundance and low-cost, lignocellulose is utilized as a good resource for producing renewable energy, feed, fertilizer, industrial raw materials, and base materials [4].

Bioethanol as transport gasoline additives can reduce carbon dioxide emissions in a certain extent. However, some researchers statistical analysis the related parameters during bioethanol production and build modeling. They found in most of bioethanol production process the residues cannot be completely utilized for energy recovery. Thus, taking full advantage of lignocelluloses becomes research hotspot [5]. In the concept of lignocellulosic biorefinery, the pentose and hexose component is an important component that can be utilized for biotransformation, and the use of physicochemical pretreatment will cause pollution problems, energy waste and inhibitors production [6]. For bioenergy generation, the cellulose and hemicellulose in lignocellulose are hydrolyzed to obtain pentose and hexose, which can be used as raw materials [7,8]. However, lignin is another major component in lignocellulose which increases the material recalcitrant and the difficulty of enzymolysis [9]. In order to make full use of lignocellulosic materials and decrease the material recalcitrance, physical or chemical pretreatment generally used to the raw material before saccharification [10,11]. Recently, the synchronous saccharification and fermentation (SSF) process [12,13] and consolidated bioprocessing (CBP), which integrated the production of enzymes, saccharification and fermentation [14,15] were focused. In these processes, the hydrolysis of lignocellulose by enzymes was critical for SSF and CBP. It has been known that cellulolytic enzymes are classified into three groups according to their effectiveness in cellulase hydrolysis: β-glucosidase, exoglucanases and endoglucanase [16]. Several different synergistic effects have been observed between different or between the same classes of cellulolytic enzymes [17]. For example, cellulose can synergistically be degraded into cellobiose and cellooligosaccharides by endoglucanases and exoglucanases, where the endoglucanase provides the exogenous glucanase with an offensive free chain. Ultimately, β-glucosidase hydrolyze soluble cellodextrins and cellobiose to produce glucose [18]. In the presence of such synergies, cellulose degradation is more efficient.

As a multimer in the lignocellulose, hemicellulose is composed of several different monosaccharides with fewer components in the middle of two layers of cellulose microfibers [19]. In contrast to cellulose, hemicellulose is branched and has a low degree of polymerization with 100 to 150 sugar residues per chain [4,20]. Based on their composition, hemicelluloses is composed of xylan, mannan, galactan, arabinose, and xyloglucan. Hemicellulose can be hydrogen bonded to cellulose and covalently linked to lignin [21]. Researches on the synergism of cellulase and other enzymes were mainly focused on improving hydrolysis efficiency. Higher proportion of xylanase normally leads to a stronger synergistic effect, especially when the substrate has a relatively higher xylan [22]. The incorporation of endoxylanase and xyloglucanases in cellulase can improve the hydrolysis of a broad range of pretreated lignocellulosic substrates, demonstrating the potential of this synergistic strategy in practical application [23].

Our previous studies showed that the synergy effect of cellulase and xylanase was effective for lignocellulosic saccharification, and the synergistic mechanism was also explored by evaluating the substrate morphology [24]. Based on these discoveries, we co-expressed the cellulase and xylanase in *Saccharomyces cerevisiae* to enhance the lignocellulose biotransformation efficiency. The recombinant *Saccharomyces cerevisiae* with co-expression of cellulase and xylanase showed better property for lignocellulose biotransformation. In this study we demonstrate a strategy of co-expression of cellulase and xylanase in *saccharomyces cerevisiae*, and use PDCS as the substrate. Comparing the enzyme activity, biomass and ethanol production between co-expression strain and single express strain, the dominance of co-expressed strains can demonstrate the importance of the synergistic effect of cellulase and xylanase. This strategy establish a foundation of the mechanism research of synergy effect of cellulase and xylanase.

## 2. Materials and methods

### 2.1. Plasmids and strains

The pHM368-pgk-TS-Ura, pHM368-pgk-CA-Ura, pHM368-pgk-CBH-Ura were constructed and preserved in our laboratory. *Escherichia coli* XL10-Gold was used for plasmid cloning was purchased from Novagen, and the *S. cerevisiae INVSc1* (*His^−^, Leu^−^, Trp^−^, Ura^−^*) was purchased from Thermo Fisher Scientific Inc.

### 2.2. Medium

The yeast extract peptone dextrose (YPD) and lysogeny broth (LB) medium used to culture and preserve *S. cerevisiae* and *E. coli*, respectively. Synthetic complete (SC) medium (0.67% yeast nitrogen base, 2% glucose, 0.01% His, 0.01% Leu and 0.01% Trp) was used for functional screening of recombinant *S. cerevisiae*. The YPC (1% yeast extract, 2% peptone, 2% Carboxymethyl cellulose-Na), the YPX (1% yeast extract, 2% peptone, 2% xylan extracted from beech), and the SC-Ura-Leu medium (0.67% yeast nitrogen base, 2% glucose, without uracil and leucine) were used for screening recombinant *S. cerevisiae*. The YPC with 0.01% Congo red and the YPX with 0.01% trypan blue medium were used for functional verification. The corn stover medium (1% yeast extract, 2% peptone, and 2.5% PDCS power) was used for fermentation.

The corn stover (CS) were collected from a local farm in Wuhan, Hubei, China. The dried CS were ground into powder and washed with deionized water until the powders without soluble components. The insoluble precipitates filtered by vacuum suction, then placed in 50°C untill dried. Lignocellulosic samples were the partly delignified corn stover (PDCS) by acid pretreatment, nitric acid and absolute ethyl alcohol with a solid to liquid ratio of 2:18.7:9.3 (w/v), and CS was placed in a 76°C water for 3 h.

### 2.3. Construction of the recombinant plasmids

The leucine primers with restriction sites *Nco*I and *Sse*8387I were designed by referencing the Leucine sequence (Genbank NO: AF063849.1), primers: Leu-f, 5ʹ-CGCCCATGGTTAAGCAAGGATTTTCT-3ʹ; Leu-r, 5ʹ-AAACCTGCAGGTCTGCC CCTATGTCTGCCCCTA-3ʹ. The leucine sequence was amplified by chain reaction with *ex-Taq* DNA polymerase (TaKaRa, Dalian, China) from pESC-Leu (Agilent Stratagene, Catalog#217452).

The amplified leucine fragments were digested with *Nco*I and *Sse*8387I (TaKaRa, Dalian, China), and cloned into pHM368-pgk-TS-Ura, pHM368-pgk-CA-Ura and pHM368-pgk-CBH-Ura was digested with the same restriction enzymes. Then the pHM368-pgk-TS-Leu, pHM368-pgk-CA-Leu and pHM368-pgk-CBH-Leu were obtained with the uracil selection marker being modified into the leucine screening marker.

### 2.4. Co-expression of cellulase and xylanase in saccharomyces cerevisiae

To prepare *S. cerevisiae* electrophoresis competent cells, a single colony of *S. cerevisiae INVSc1* was inoculated into 50 mL YPD liquid medium at 28°C for 18 h. Then the cell culture was inoculated into 100 mL fresh YPD medium with an initial OD_600_ of 0.3 at 28°C. After cultured to an optical density at 600 nm (OD_600_) of 1.0, the cells were collected in a 50-mL centrifuge tube, and pre-cooled sorbitol resuspended cells were further resuspended in a final volume of 1 to 1.5 mL. The linearized plasmids DNA digested with *Hpa* I was added to 100 μL of the competent cells, followed by mixing and conversion at 1.5 KV using an electric transducer (Bio-Rad MicroPulser, USA). Then, the transformants were screened using SC plates. Specifically, *S. cerevisiae* strains that simultaneously transferred and expressed the cellulase gene and the xylanase gene were screened on the SC-Ura-Leu plate.

The ability of the recombinant strain to express cellulase was verified using a CMC Congo red plate. A single colony from the auxotrophic SC plate was placed on the YPD plate and incubated at 28°C to promote its growth. The single colony of recombinant *S. cerevisiae INVSc1*-CBH-CA and *INVSc1*-CBH-TS were selected from the YPD solid plate, transferred to a YPD plate containing 1% CMC, and incubated at 28°C for several days. When the colony growth was sufficient to clean the colony, it was sprayed with red dye solution, left for 15–20 min, and then rinsed with sodium chloride (NaCl) to observe the yellow bacteria circle, which verified the expression of cellulase.

The xylan-trypan blue plate was used to verify the ability of recombinant strains to express xylanase. The single colony from the auxotrophic SC plate was selected and transferred to the YPD plate, which was incubated at 28°C to promote its growth. The single colony of recombinant *S. cerevisiae INVSc1*-CBH-TS, *INVSc1*-CBH-CA were transferred into trypan blue medium for 4 to 5 days to confirm the xylanase expression and activity of the recombinant strain.

### 2.5. Inoculum preparation and enzyme assays

The recombinant single colony strain was inoculated into a conical flask containing 50 mL YPD and cultured at 28°C with agitation at 200 rpm until the yeast grew to an OD_600_ of 2.0. The supernatant was collected by centrifugation at 4000 rpm for 1 min and used as the enzyme solution.

Specifically, 0.6 mL enzyme solution was added to a 50 mL colorimetric tube and pre-heated for 5 min in a 50°C water bath for 30 min after adding 1.4 mL CMC-Na (or xylan) solution. Then 2.0 mL of a 2 mol/L sodium hydroximde (NaOH) solution and 3.0 mL DNS were added, followed by boiling in a water bath for 5 min to terminate the reaction. After cooling the solution in a water bath, the absorbance was measured at 540 nm. 2.0 mL of a 2 mol/L NaOH solution to inactivate the enzyme fluid operation was used as the control. The measurements were performed in three independent times. The enzyme activity was defined as the amount of enzyme that released 1 μg of glucose (or xylose) per hour for one enzyme activity unit (U) under the above conditions.

### 2.6. Growth and fermentation of recombinant S. cerevisiae

The recombinant yeast and *INVSc1* were transferred to 1 mL YPD liquid medium with PDCS as the sole carbon source and cultured at 28°C with agitation at 200 rpm. After 24 h cultivation, the samples were diluted 10^5^ times and plated on YPD plates, and then the number of grown colonies was counted after culturing at 28°C for 24 h.

The recombinant yeast and *INVSc1* were inoculated into 10 mL liquid YPD medium with PDCS as the sole carbon source and static cultured for fermentation. The samples were incubated and the ethanol concentration in the sample solution was measured using a biosensor (Biosensors glucose analyzer, Sieman Technology Co, Ltd) with ethanol enzymatic membrane at 24, 48, 72, 96, 120 and 144 h. The biosensor was calibrated with standard ethanol concentration before used to measure the ethanol concentration.

## 3. Results

### 3.1. Co-expression of cellulase and xylanase in S. cerevisiae

**Figure 1.**
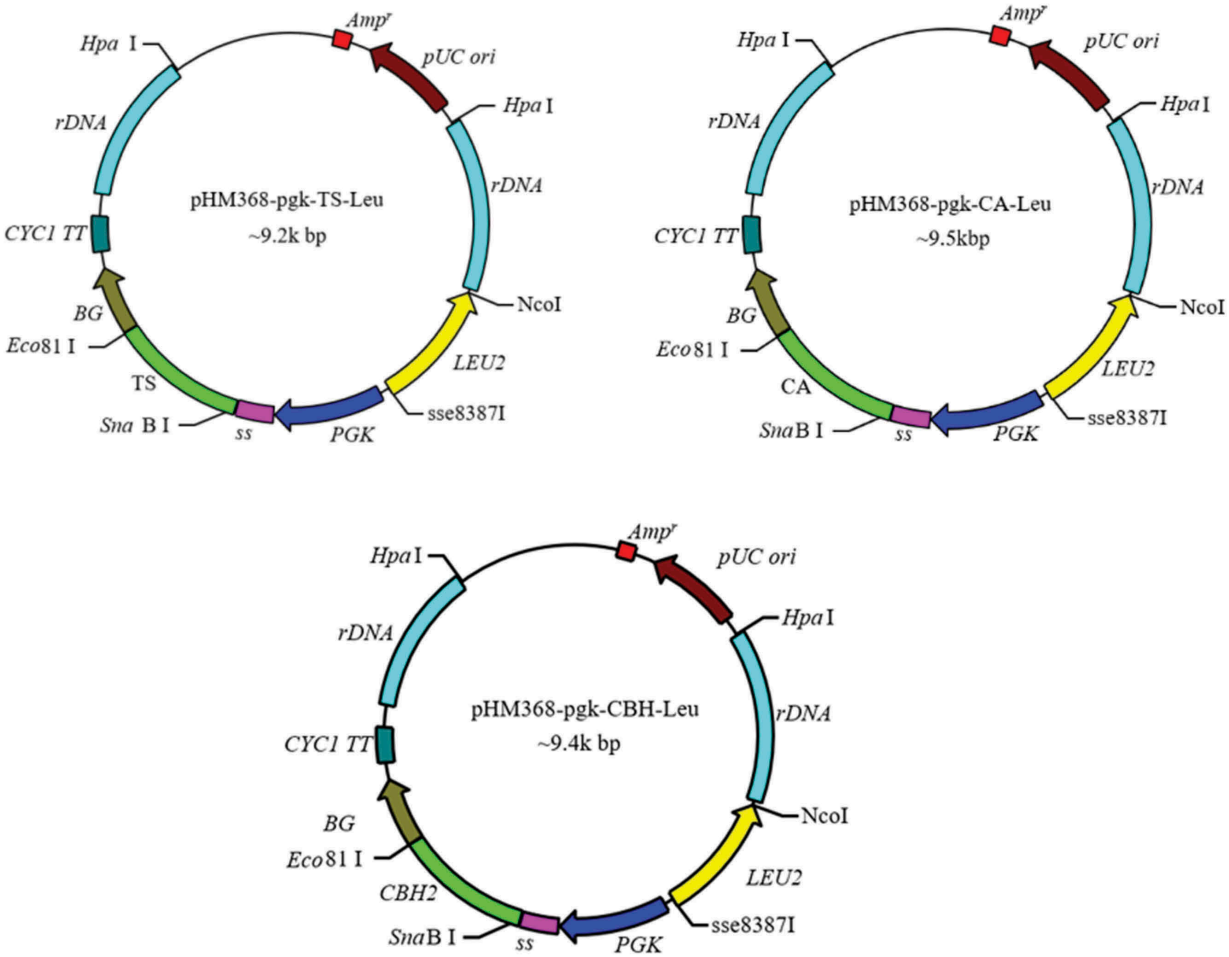
The recombinant plasmids of pHM368-pgk-TS-Ura, pHM368-pgk-CA-Ura and pHM368-pgk-CBH-Ura in this work.

The recombinant plasmids (Figure 1) were linearized with *Hpa* I and mixed in molar ratios of 1:1 to form four combinations of cellulase and xylanase genes. The mixed linearized plasmids were electro-transformed into *S. cerevisiae INVSc1* and then screened by dual auxotrophic screening medium SC-Ura-Leu plates to obtain recombinant strains co-expressing cellulases and xylanases.

**Figure 2.**
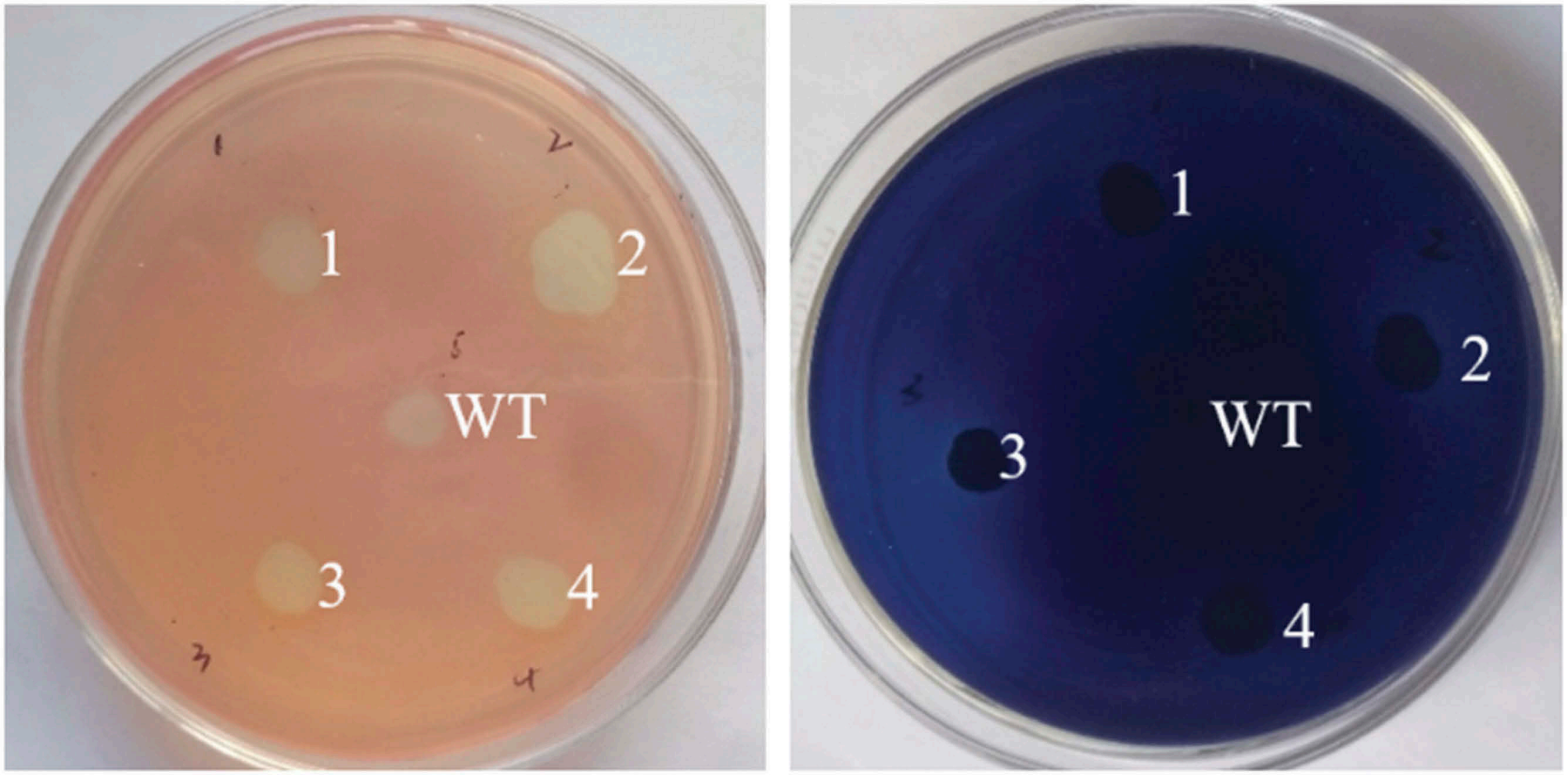
Functional verification of the recombinant xylanase and cellulase. Blue and red indicate verification of xylanase and cellulase using trypan blue and congo red plates, respectively. WT, *S. cerevisiae INVSc1*; 1–4, the recombinant *S. cerevisiae* with xylanase and cellulase activities.

The function of screened strains were validated by CMC-Congo red plate and Xylan-trypan blue plate (Figure 2). The results showed that the cellulase and xylanase genes were successfully co-expressed in *S. cerevisiae*. Two recombinant *S. cerevisiae* with high cellulase and xylanase activities were selected and named as *INVSc1*-CBH-CA and *INVSc1*-CBH-TS, respectively. At the same time, the strain express single cellulase or xylanase were also verified and named as *INVSc1*-CBH, *INVSc1*-CA and *INVSc1*-TS.

The cellulase and xylanase activity of the recombinant *S. cerevisiae* were evaluated by dinitrosalicylic acid (DNS) method. The cellulase activity reached 931.27 U/mL, and the xylanase activity reached 413.70 U/mL in *INVSc1*-CBH-CA. While in *INVSc1*-CBH-CA, the cellulase activity reached 716.43 U/mL, and the xylanase activity reached 205.13 U/mL.

### 3.2. Recombinants growth and fermentation using PDCS as sole carbon source

**Figure 3.**
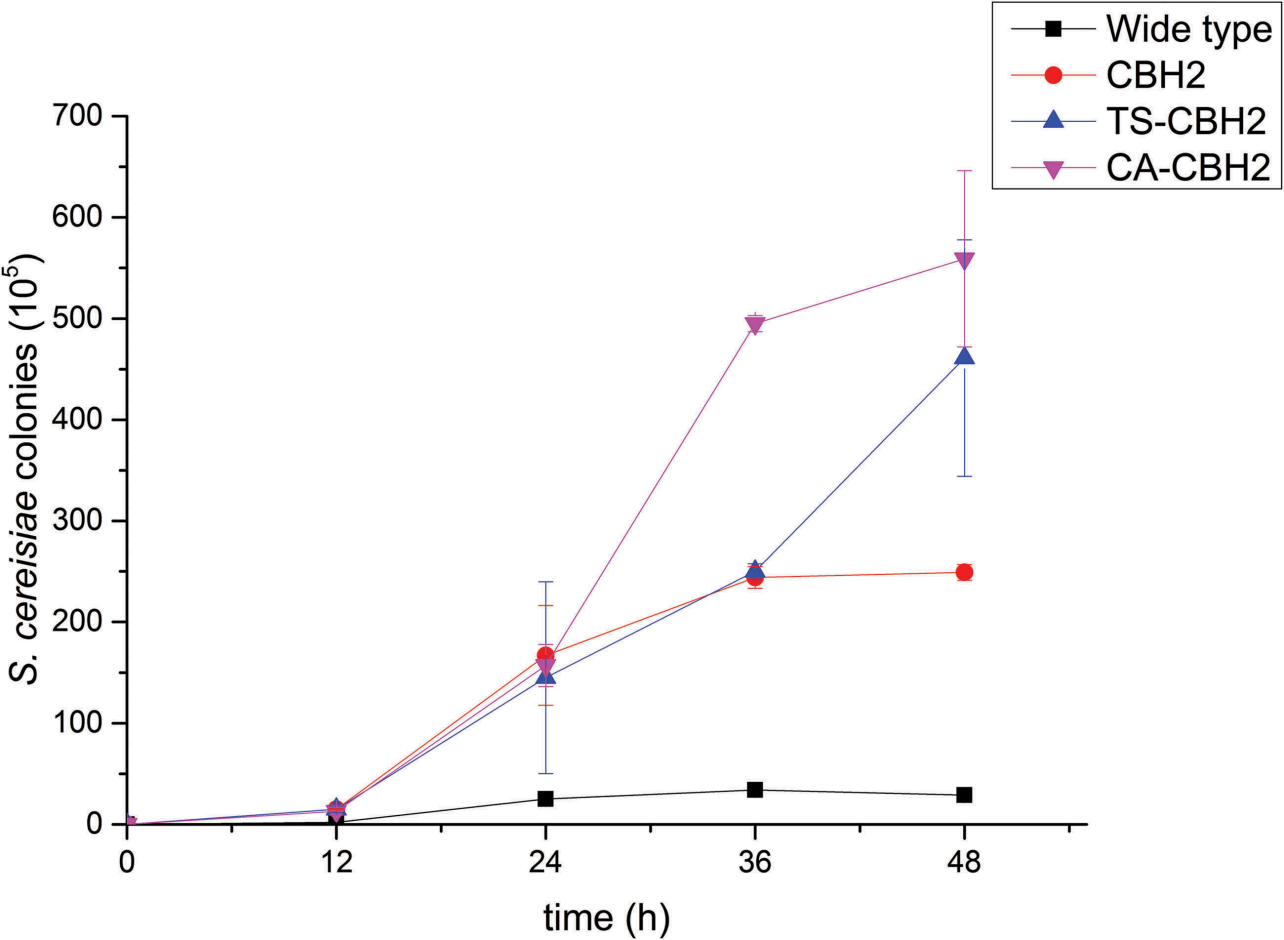
The ethanol yields of different recombinant *S. cerevisiae* during the fermentation.

*INVSc1*-CBH, *INVSc1-*CA, *INVSc1-*TS, *INVSc1-*CBH-CA, and *INVSc1-*CBH-TS were cultured with PDCS as the sole carbon source to evaluate the PDCS utilizing ability. The *S. cerevisiae INVSc1* was used as a control, and the ability of utilizing PDCS was evaluated by microbial count method. The results showed that the *INVSc1*-CBH-CA and *INVSc1*-CBH-TS can make full use of PDCS to grow, with the better efficiency than the *INVSc1*-CBH, while the *S. cerevisiae INVSc1* scarcely grows. It can be speculated that co-expressing xylanase and cellulases improve the PDCS enzymolysis and provide more available carbon source for the *S. cerevisiae* growing (Figure 3).

**Figure 4.**
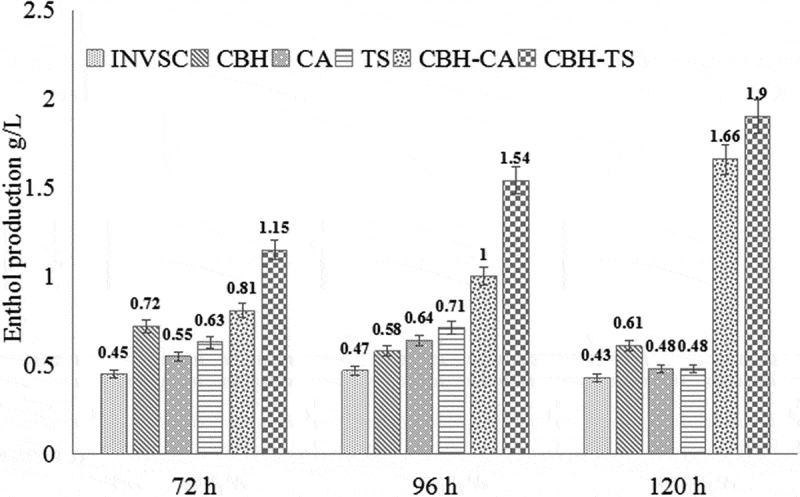
Comparison of the ethanol yields among different recombinant *S. cerevisiae.*

In addition, fermentation of the recombinant *S. cerevisiae* using PDCS as the sole carbon source were also carried out as described in methods. The ethanol production during the fermentation with the different recombinant *S. cerevisiae* was evaluated. The ethanol yields of *INVSc1*-CBH-CA and *INVSc1*-CBH-TS reached the highest of 1.66 g/L and 1.90 g/L after 120 h cultivation, which were much higher than other recombinant *S. cerevisiae* (0.48–0.61 g/L ethanol) and *INVSc1* (0.43 g/L ethanol) (Figure 4). At 72 h, the ethanol yield of *INVSc1*-CBH-CA increased by 0.1 and 0.8 folds higher than that of *INVSc1*-CBH and *S. cerevisiae INVSc1*, respectively. Comparably, the ethanol yield of *INVSc1*-CBH-CA was nearly 0.6 folds higher than that of *INVSc1-CBH*, and 1.6 folds higher than that of *S. cerevisiae INVSc1* at 72 h. At 96h, the ethanol yields of *INVSc1*-CBH-CA and *INVSc1*-CBH-TS were 0.7 and 1.7 folds higher than *S. cerevisiae INVSc1-*CBH, 1.1 and 2.3 folds higher than *S. cerevisiae INVSc1*, respectively. Finally at 120 h, the ethanol yields of *INVSc1*-CBH-CA and *INVSc1*-CBH-TS were 1.7 and 2.1 folds higher than *S. cerevisiae INVSc1-*CBH, 2.8 and 3.4 folds higher than *S. cerevisiae INVSc1*. In addition, when comparing the cellulase and xylanase co-expressed strains with the strains that separately expressed cellulase or xylanase, the ethanol production of *INVSC1*-CBH-CA and *INVSC1*-CBH-TS at 96 h and 120 h (1.00g/L, 1.54g/L and 1.66g/L, 1.90g/L) was higher than *INVSC1*-CBH and *INVSC1*-CA or *INVSC1*-TS (0.58 + 0.64g/L, 0.58 + 0.71g/L and 0.61 + 0.48g/L, 0.61 + 0.48g/L) which indicates the synergistic effect between co-expressed strains. As shown in Figure 4, the recombinant *S. cerevisiae* co-expressing cellulase and xylanase can utilize PDCS more efficiently and produce more ethanol than the *S. cerevisiae* only expressing cellulase. These results indicate that the synergistic effect of cellulase and xylanase improve the saccharification efficiency of lignocellulose and increase the ethanol yield of *S. cerevisiae* during fermentation. This probably due to the spatial structure of xylan and cellulose in lignocellulosic materials, which in our previous work, we used SEM to observe the material after xylanase hydrolysis [24]. Because of the complicated cross-linking between cellulose and xylan, the xylanase could break down the network structure of lignocellulosic substrates to increase swelling and porosity. When the engineered *S. cerevisiae* co-expressed cellulase and xylanase, the contact area of cellulose increased, and the accessibility of cellulase increased, so that more monosaccharides could be hydrolyzed for the growth of strains and biological transformation of ethanol.

## 4. Discussion

In the results, it demonstrate that co-expressing cellulases and xylanases increase the ability of *S. cerevisiae* to produce ethanol. During the biohydrolysis of lignocellulose, xylanase promoted the hydrolysis of xylan cross-linked cellulose in lignocellulose structures, while simultaneously generated xylo-oligosaccharides and D-xylose. The lignocellulosic materials became perforated, peeled, and fractured tubular structures became loose after the lignocellulose substrate was hydrolyzed by xylanase. The accessibility of cellulose increased and the cellulase not only hydrolyze the exposed cellulose components, but also the part of cellulose that was encapsulated inside, thus enhancing the contact area of cellulose enzymes in the reaction with cellulose. Moreover, the recombinant *S. cerevisiae INVSc1, INVSc1*-TS, and *INVSc1*-CA can use corn stover as the sole carbon source to produce a certain amount of ethanol. We analyze the presence of a small amount of reducing sugars in the medium with corn stover as the sole carbon source. When the medium was sterilized at a high temperature of 108°C, a certain amount of reducing sugars will be produced due to the high temperature effect of lignocellulose. As a result, the recombinant *S. cerevisiae INVSc1, INVSc1*-TS, and *INVSc1*-CA strains utilize a small amount of reducing sugar to produce ethanol.

In recent studies on the co-expression of xylanase and cellulose, a part of studies focused on the enzymatic properties and product analysis of heterologous expression xylanase. Bifunctional CtCel5E was fused with a β-glucosidase from *Clostridium cellulovorans* (CcBglA), the fusion expression improved the enzyme stability and glucose yield [25]. The analysis of the enzymatic hydrolysis products of heterologous expression xylanase is also an important research focus [26,27]; The other part focuses on the synergistic effect of recombinant expressed xylanase and commercial cellulase, such as two different sources of xylanase (rXYN10A MALCI and rXYN11A MALCI) act synergistically with commercial cellulases and resulted in 1.54 and 1.58 folds improved hydrolysis of acid treated rice straw and alkali treated rice straw [28]. In another research, two different sources of xylanase (rXyn162 and rXylCD2) exhibited synergistic hydrolysis of oat spelts xylan and sodium hydroxide pretreated corn stover (SHPC). These xylanase could efficiently improve the hydrolysis of SHPC by commercial cellulase [29]. Moreover, Ming-Hsu Chen et al. discussed the synergistic effect of cellulase and xylanase from the SEM of material structure and composition analysis, and the result show that the synergistic effect could reduce the amount of cellulase added in the saccharification process [30]. Other studies have focused on sugar production during saccharification, such as the reconstructed *A. niger* cellulase could hydrolyze wheat straw to release 19.70 g/L of glucose, compared to 12.01 g/L of that released by the cellullase from the original *A. niger* [31]. However, the promotion of ethanol yield from the synergistic effects of cellulase and xylanase is not discussed in most studies, thus we focus on the effect of synergies on ethanol yield in this work. The ethanol yield of *INVSc1*-CBH-CA and *INVSc1*-CBH-TS compared to *INVSc1*-CBH, *INVSC1*-CA and *INVSC1*-TS. When pretreated corn stover was used as the sole carbon source, *INVSc1*-CBH-CA and *INVSc1*-CBH-TS ethanol yields increased by 1.7 and 2.1 folds higher than *INVSc1*-CBH, 2.8 and 3.4 folds higher than the wild type *S. cerevisiae*. The strategy of co-expression cellulase and xylanase in *saccharomyces cerevisiae* is effective and the ethanol yield of the recombinant strain co-express cellulase and xylanase was increased.

## 5. Conclusions

In our studies, cellulase and xylanase were successfully co-expressed in *S. cerevisiae* with their functions being verified. The activity of cellulase and xylanase reached 931.27 U/mL and 413.70 U/mL in *INVSc1*-CBH-TS and 716.43 U/mL and 205.13 U/mL in *INVSc1-*CBH-CA. Both the recombinant *INVSc1*-CBH-CA and *INVSc1*-CBH-TS can use PDCS as the sole carbon source efficiently for ethanol production, and achieving the highest yields of 1.66 g/L and 1.90 g/L after cultivation of 120 h. Our studies provided evidence that co-expressing cellulase and xylanase in *S. cerevisiae* can improve the lignocellulose biotransformation efficiently.

## Highlights

- Co-expression of cellulose and xylanase in *Saccharomyces cerevisiae*;
- Production of bioethanol by corn stover; Synergistic action of cellulase and xylanases in *Saccharomyces cerevisiae*.

## References

[1] Gray KA, Zhao L, Emptage M. Bioethanol. Curr Opin Chem Biol. 2006;10(2):141–146. 16522374 10.1016/j.cbpa.2006.02.035

[2] Howard RL, Abotsi E, Van Rensburg EL, et al Lignocellulose biotechnology: issues of bioconversion and enzyme production. Hepatobiliary Pancreatic Dis Int. 2003;2(12):602–619.

[3] Liu Y, Chen W, Xia Q, et al Efficient cleavage of lignin–carbohydrate complexes and ultrafast extraction of lignin oligomers from wood biomass by microwave‐assisted treatment with deep eutectic solvent. ChemSusChem. 2017;10(8):1692–1700. 28054749 10.1002/cssc.201601795 PMC5413814

[4] Xia Q, Chen Z, Shao Y, et al Direct hydrodeoxygenation of raw woody biomass into liquid alkanes. Nat Commun. 2016;7:11162. 10.1038/ncomms11162 PMC4820995 27025898

[5] Zhao Y, Damgaard A, Xu Y, et al Bioethanol from corn stover – global warming footprint of alternative biotechnologies. Appl Energy. 2019;247:237–253.

[6] Chandel AK, Garlapati VK, Singh AK, et al The path forward for lignocellulose biorefineries: bottlenecks, solutions, and perspective on commercialization. Bioresour Technol. 2018;S0960852418307831. 10.1016/j.biortech.2018.06.004 29960825

[7] Mussatto SI, Fernandes M, Milagres AMF, et al Effect of hemicellulose and lignin on enzymatic hydrolysis of cellulose from brewer’s spent grain. Appl Biochem Biotechnol. 2008;43(2):124–129.

[8] Shen Z, Zhang K, Si M, et al Synergy of lignocelluloses pretreatment by sodium carbonate and bacterium to enhance enzymatic hydrolysis of rice straw. Bioresour Technol. 2018;249:154–160. 10.1016/j.biortech.2017.10.008 29040849

[9] Mikhail B, Ewellyn C, Hanna G, et al Quantification of lignin-carbohydrate linkages with high-resolution NMR spectroscopy. Planta. 2011;233(6):1097–1110. 10.1007/s00425-011-1359-2 21298285

[10] Ye S, Jiayang C Hydrolysis of lignocellulosic materials for ethanol production: a review. Bioresour Technol. 2003;83(1):1–11. 10.1016/s0960-8524(01)00212-7 12058826

[11] Weil JR, Dien B, Bothast R, et al Removal of fermentation inhibitors formed during pretreatment of biomass by polymeric adsorbents. Ind Eng Chem Res. 2002;41(24):6132–6138.

[12] Chen Y, Liu Q, Zhou T, et al Ethanol production by repeated batch and continuous fermentations by Saccharomyces cerevisiae immobilized in a fibrous bed bioreactor. J Microbiol Biotechnol. 2013;23(4):511–517. 10.4014/jmb.1209.09066 23568205

[13] Song H, Liu J, Yang L, et al Genetically modified Saccharomyces cerevisiae for one-step fermentation of bioalcohol using corncob as sole carbon source. Ann Microbiol. 2014;64(2):781–785.

[14] Song HT, Liu SH, Gao Y, et al Simultaneous saccharification and fermentation of corncobs with genetically modified Saccharomyces cerevisiae and characterization of their microstructure during hydrolysis. Bioengineered. 2016;7(3):198–204. 10.1080/21655979.2016.1178424 PMC4927203 27116398

[15] Olson DG, Mcbride JE, Shaw AJ, et al Recent progress in consolidated bioprocessing. Curr Opin Biotechnol. 2012;23(3):396–405. 10.1016/j.copbio.2011.11.026 22176748

[16] Engels B, Dahm P, Jennewein S Metabolic engineering, metabolic engineering of taxadiene biosynthesis in yeast as a first step towards Taxol (Paclitaxel) production. Metab Eng. 2008;10(3):201–206. 18485776 10.1016/j.ymben.2008.03.001

[17] Gusakov AV, Kondratyeva EG, Sinitsyn AP Comparison of two methods for assaying reducing sugars in the determination of carbohydrase activities. Int J Anal Chem. 2011;2011(1687–8760):283658. 10.1155/2011/283658 PMC3103847 21647284

[18] Asztalos A, Daniels M, Sethi A, et al A coarse-grained model for synergistic action of multiple enzymes on cellulose. Biotechnol Biofuels. 2012;5(1):55. 10.1186/1754-6834-5-55 PMC3475064 22853643

[19] Sjostrom E Wood chemistry: fundamentals and applications. Academic Press. ISBN 9780080925899. 1993;1–20..

[20] Chen F, Lu Z Liquefaction of wheat straw and preparation of rigid polyurethane foam from the liquefaction products. J Cell Biochem. 2010;111(1):508–516.20568118

[21] Schadel C, Bochl A, Richter A, et al Quantification and monosaccharide composition of hemicelluloses from different plant functional types. Plant Physiol Biochem. 2010;48(1):1–8. 10.1016/j.plaphy.2009.09.008 19926487

[22] Jinguang H, Richard C, Valdeir A, et al The addition of accessory enzymes enhances the hydrolytic performance of cellulase enzymes at high solid loadings. J Bioresour Technol. 2015;186:149–153. 10.1016/j.biortech.2015.03.055 25812819

[23] Hu J, Arantes V, Pribowo A, et al The synergistic action of accessory enzymes enhances the hydrolytic potential of a “cellulase mixture” but is highly substrate specific. Biotechnol Biofuels. 2013;6(1):112. 10.1186/1754-6834-6-112 PMC3750293 23915398

[24] Song HT, Gao Y, Yang YM, et al Synergistic effect of cellulase and xylanase during hydrolysis of natural lignocellulosic substrates. Bioresour Technol. 2016;219:710–715. 27560367 10.1016/j.biortech.2016.08.035

[25] Chen C, Yao J, Yang B, et al Engineer multi-functional cellulase/xylanase/β-glucosidase with improved efficacy to degrade rice straw. Bioresour Technol Rep. 2019;5:170–177.

[26] Liew KJ, Ngooi CY, Shamsir MS, et al Purification, heterologous expression, purification and biochemical characterization of a new endo-1, 4-β-xylanase from Rhodothermaceae bacterium RA. Protein Expr Purif. 2019;105464. 10.1016/j.pep.2019.105464 31376486

[27] Liang W, Liu TC, Chang C, et al Bioactivity of β-1,3-xylan extracted from caulerpa lentillifera by using escherichia coli clearcoli BL21(DE3)-β-1,3-xylanase XYLII. J Food Nutr Res. 2015;3(7):437–444.

[28] Basotra N, Joshi S, Satyanarayana T, et al Expression of catalytically efficient xylanases from thermophilic fungus Malbranchea cinnamomea for synergistically enhancing hydrolysis of lignocellulosics. Int J Biol Macromol. 2018;108:185–192. 29174359 10.1016/j.ijbiomac.2017.11.131

[29] Zhuo R, Yu H, Qin X, et al Heterologous expression and characterization of a xylanase and xylosidase from white rot fungi and their application in synergistic hydrolysis of lignocellulose. Chemosphere. 2018;212:24–33. 30138852 10.1016/j.chemosphere.2018.08.062

[30] Chen MH, Kim SM, Raab RM, et al Heterologous expression of thermoregulated xylanases in switchgrass reduces the amount of exogenous enzyme required for saccharification. Biomass Bioenergy. 2017;107:305–310.

[31] Xue D, Jiang Y, Gong C Exogenous xylanase expression simultaneously with the indigenous cellulase to increase the cellulose hydrolysis efficiency. Int Biodeterioration Biodegrad. 2019;140:126–132.

